# Rapidly progressive osteoarthritis of hip: establishing and validating diagnostic criteria in the Southeast Asian population

**DOI:** 10.1186/s42836-021-00107-2

**Published:** 2022-02-03

**Authors:** Lok-chun Man, Yip-kan Yeung, Sheung-tung Ho, Ming-yu Chiu, Nin-yuan Pan

**Affiliations:** 1 Department of Orthopaedics and Traumatology, Caritas Medical Centre, 111 Wing Hong Street, Hong Kong, China; 2 Department of Radiology, Caritas Medical Centre, 111 Wing Hong Street, Hong Kong, HKSAR China

**Keywords:** Rapidly progressive arthritis, Dysplasia, Osteoarthritis, Osteonecrosis

## Abstract

**Background:**

The study aimed to establish quantitative diagnostic criteria for rapidly progressive osteoarthritis (RPOA) of the hip and to compare the criteria with those for other pathological hip entities in the Asian population.

**Methods:**

From July 2011 to September 2019, 126 patients who had undergone hip replacement were retrospectively recruited from a fast-track joint replacement list. Patient demographics and radiological parameters were evaluated. Diagnosis of hip RPOA was established based on Lequesne *et al*’s criteria. The patients with RPOA, hip dysplasia, avascular necrosis, and primary osteoarthritis were allocated to the corresponding groups separately and compared. The diagnostic criteria of RPOA were established and validated in the sample population.

**Results:**

Diagnosis of hip RPOA was confirmed in 18 patients. Their mean age at surgery (72 years) was significantly higher in this group than in the dysplasia and avascular necrosis groups. The mean pelvic tilt parameter (0.485) of RPOA group was significantly lower than those of other groups. The mean initial Tonnis angle (8.35°) of RPOA group was significantly higher than those of avascular necrosis and osteoarthritis groups. The differences were statistically significant between RPOA and non-RPOA groups in limb shortening rate, superior joint space narrowing, acetabular destruction, and head destruction (*P* < 0.05). Tonnis angle and lateral subluxation also increased significantly during the disease progression.

**Conclusion:**

Posterior pelvic tilt and increased Tonnis angle may contribute to the pathogenesis of RPOA, leading to progressive acquired acetabular obliquity and lateral subluxation. We propose the modern comprehensive diagnostic criteria be based on the existing literature and the current findings. Further external validation is recommended.

## Introduction

Rapidly progressive osteoarthritis (RPOA) of the hip, also known as rapidly destructive osteoarthritis of hip, is a rare pathological condition manifesting as rapid chondrolysis (Type 1 RPOA) followed by hip joint destruction (Type 2 RPOA). The reported incidence of RPOA is 16, and 10% of total hip arthroplasties fulfilled the diagnosis of RPOA [1, 2]. Though the disease entity has been described by multiple studies, the quantitative diagnostic criteria have not yet been reported.

RPOA was first described in 1957 [3]. Lequesne *et al* [4] proposed the first and most popularly adopted diagnostic criteria, including progressive chondrolysis exceeding 2 mm per year or the loss of more than 50% joint space within 1 year. Meanwhile, other causes of rapidly destructive conditions, such as avascular necrosis (AVN), Charcot neuroarthropathy and infection are excluded. The diagnosis established based on these diagnostic criteria entails observation for a long period of time. In some patients, however, rapid chondrolysis may develop, with subsequent destruction of the femoral head and acetabulum within 12 months following initial presentation [5, 6]. It is controversial whether bone destruction occurs as the late sequelae of cartilage destruction, or it is another disease entity distinctly different from RPOA without bone destruction.

In order to facilitate a timely diagnosis without necessitating 12-month serial follow-ups, Zazgyva *et al* [2] proposed clinico-radiological descriptive criteria for RPOA, emphasizing the presence of subchondral cysts (geodes) in the acetabulum and femoral head with a relative absence of osteophytes as the hallmark features of RPOA. The clinical features include 3 years of preceding hip pain which deteriorates in the recent 6 months, while hip mobility is relatively spared. Age, BMI, and biological factors, such as the elevated serum MMP-3 concentration, have also been mentioned in clinical description [7, 8]. The histological assessments of RPOA specimens include degenerative fibrosis, hyalinization, and chrondromatosis, associated with synovial hyperplasia and chronic perivascular inflammation [2]. Although the destructive processes can be used to distinguish RPOA from primary osteoarthritis (OA), the assessments are complex and costly. Until now, the incidence has not been reported in Asian countries, and only one study in Japan explored the disease at the early stage [7].

The study aimed to introduce novel quantitative diagnostic criteria for hip RPOA and validate the efficacy of the criteria by comparing it with other pathological hip entities. To our knowledge, it was the first reported scoring-based system in the literature and the only cohort series for advanced disease progression in the Asian population.

## Materials and methods

The research project was approved by the Research Ethics Committee, Kowloon West Cluster, Hong Kong (Study Code: KW/EX-21-058(158–01)). Informed consent was waived.

### Patient selection

We conducted a retrospective longitudinal review of a group of patients recruited from our fast-track joint replacement list. All hip replacement operations were performed in the single hospital from July 2011 to September 2019. The patients’ medical history, clinical manifestations, radiological features, and culture and pathological specimen findings of the identified cases were reviewed. Diagnosis of RPOA was made on the basis of the aforementioned criteria. Radiographically suspicious RPOA was further studied in conjunction with a radiologist. Diagnoses other than RPOA were categorized into (1) hip dysplasia with an initial Wiberg angle of less than 25 degrees, or a Tonnis angle of more than 10 degrees [9]; (2) radiographic AVN characterized by femoral head lucency, sclerosis, or flattening before joint space narrowing; and (3) primary osteoarthritis with osteophytes and gradual joint space narrowing as the predominant radiological features.

The inclusion criteria were: (1) patients who had a history of rapid clinical deterioration of hip joint condition with a significant bone loss or deformity; (2) functional impairment of the hip joint that mainly affected independence or occupation/performance of social role, and (3) patients met the recommendations from the Central Coordinating Committee in our locality. The exclusion criteria included: (1) an established diagnosis of infection, neuropathic arthropathy, post-traumatic and congenital deformities; (2) inflammatory conditions, autoimmune, and endocrine conditions; (3) patients with incomplete imaging sequences; and (4) stable cases with no significant functional or radiological deterioration. In patients with bilateral hip involvement, the clinical and radiological progression of the firstly presented hip, usually being the more severely affected hip, would first be assessed. The contralateral hip would then be assessed following fast-track operation of the firstly presented hip.

### Review method

**Fig. 1:**
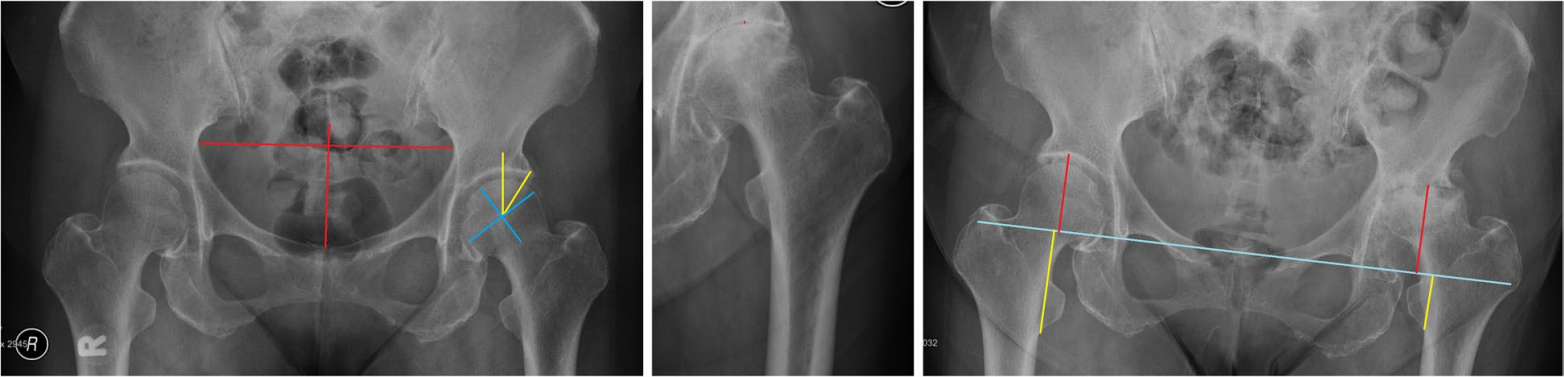
Left (normal), measuring the pelvic tilt diameter, Wiberg angle, and head diameter. Middle (type 1 RPOA, loss of joint space), measuring the superior joint space. Right (type 2 RPOA, superior acetabulum and femoral head destruction), measuring the superior acetabular bone loss and final leg length discrepancy

**Fig. 2:**
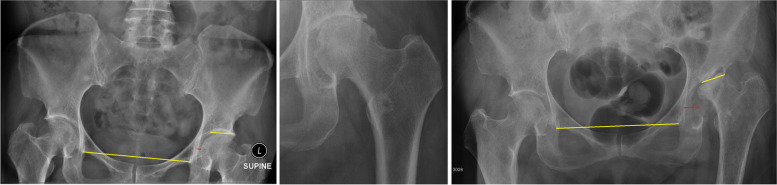
Measuring the changes of Tonnis angle and lateral subluxation. The Tonnis angle and lateral subluxation progressed within 9 months with radiological progression from normal (left) to type 1 (middle) and type 2 (right) RPOA. The Tonnis angle increased from 5 to 11 degrees, and lateral subluxation from 5 to 17 mm

We reviewed the standard supine antero-posterior images of the pelvis centered over the pubic symphysis. We compared the radiographic features between the first available film and the immediate preoperative film (Figs. 1 and 2). We defined leg length discrepancy as the length difference between the two hips, measured longitudinally from the teardrop line to the lessor trochanter. The Wiberg angle was measured between the vertical line through the center of femoral head, and the line drawn from the hip center to the lateral acetabular rim. The Tonnis angle was measured between the horizontal inter-teardrop line and the line tangential to the weight-bearing sourcil. Pelvic tilt was estimated by the ratio between vertical and horizontal diameters of the pelvic foramen [7]. Lateral subluxation was defined as the horizontal distance between the teardrops and medial head borders.

The rates of joint space and bony destruction were also analyzed, including: (1) superior acetabular bone loss measured from the teardrop line to the superior acetabular sourcil [8]; (2) acetabular volumetric widening, measured from the infero-medial to super-lateral acetabulum rim; and (3) femoral head size indicated by two lines intersecting at the head center, one perpendicular (horizontal diameter) and another parallel (vertical diameter) to the neck axis, measured down to the head-neck junction covering the imaginary head area with cartilage cover (Fig. 1).

### Statistical analysis

For *P* value calculation, we used a two-tailed *t*-test for continuous variables and Fisher’s test for categorical variables. The review and diagnosis processes were conducted by the conjoint panel, including 3 orthopedic surgeons and 1 radiologist, with at least 15-year experience in their specialty fields.

### Establishment of RPOA diagnostic criteria (Table 1)

All 18 dysplasia hips satisfied the first RPOA diagnostic criteria for exclusion. The scores were estimated in the remaining hips. Our RPOA radiological criteria presented below (Tables 2 and 3) were in consistent with the literature descriptions. There existed statistically significant differences in the measurements between RPOA group and non-RPOA groups (Table 4). Double points were awarded to each of the following parameters:

1. Initial pelvic tilt parameter of 0.485, comparable with 0.492 from Yasuda [7];
2. Initial Tonnis angle of 8.35, a finding consistent with Nelson’s study, showing significant differences between RPOA group and non-RPOA groups, with significantly lower values in the non-RPOA groups [10];
3. Annual superior joint space narrowing of 2.44 mm, in consistency with Lequesne’s finding [4];
4. Annual superior acetabular bone loss of 7.24 mm, being consistent with Karayiannis’s description of bone loss of more than 5 mm per year [8].

**Table 1:**
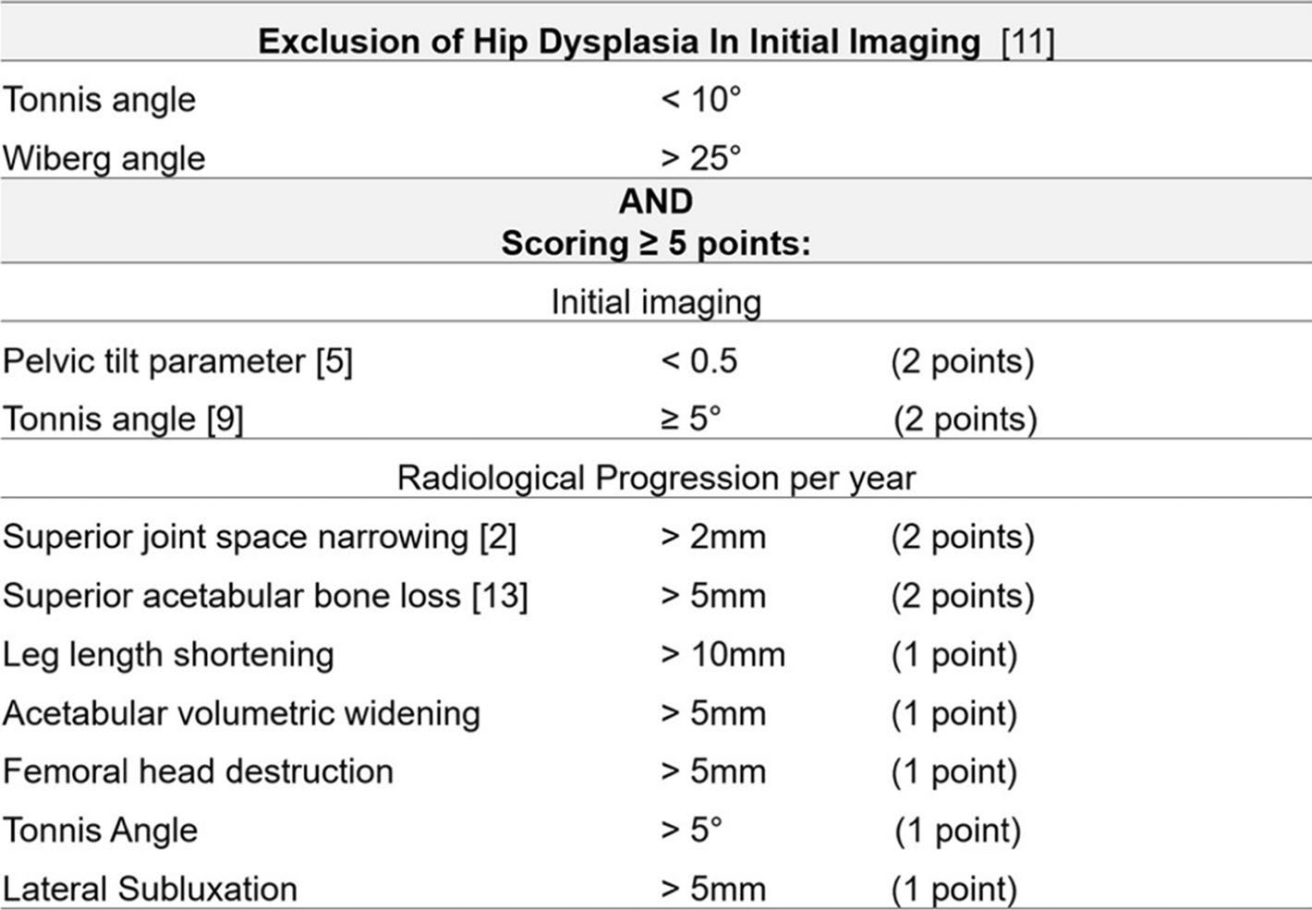
The proposed rapidly progressive osteoarthritis diagnostic criteria

**Table 2:** Clinical and radiological features of rapidly progressive osteoarthritis of 81 hips. Values are represented as mean ± standard deviation

	RPOA	Dysplasia	OA	AVN
Age at operation	72.1 ± 7.80	62.6 ± 6.27 (*P* = 0.001*)	67.4 ± 11.7 (*P* = 0.208)	55.1 ± 9.51 (*P* = 0.000*)
Sex (Male: Female)	6: 10	3: 12 (*P* = 0.433)	3: 11 (*P* = 0.440)	19: 8 (*P* = 0.055)
Side (Left: Right)	7: 11	9: 9 (*P* = 0.738)	6: 9 (*P* = 1.000)	17: 13 (*P* = 0.372)
LLD on initial film (mm)	1.67 ± 2.74	8.29 ± 7.23 (*P* = 0.002*)	3.05 ± 3.53 (*P* = 0.255)	5.53 ± 5.07 (*P* = 0.009*)
LLD on final film (mm)	21.5 ± 9.37	12.6 ± 11.6 (*P* = 0.016*)	6.14 ± 3.48 (*P* = 0.000*)	7.98 ± 7.49 (*P* = 0.000*)
Pelvic tilt	0.485 ± 0.133	0.638 ± 0.092 (*P* = 0.001*)	0.597 ± 0.129 (*P* = 0.023*)	0.637 ± 0.090 (*P* = 0.000*)
Wiberg angle (degree)	36.4 ± 8.84	14.9 ± 5.65 (*P* = 0.000*)	34.4 ± 7.08 (*P* = 0.487)	38.1 ± 6.84 (*P* = 0.470)
Tonnis angle (degree)	8.35 ± 3.35	16.6 ± 4.91 (*P* = 0.000*)	5.27 ± 4.96 (*P* = 0.046*)	4.23 ± 4.08 (*P* = 0.001*)

*LLD* leg length discrepancy, *OA* osteoarthritis, *AVN* avascular necrosis

*: statistically significant

**Table 3:** Radiological progression of joint space changes and bone destruction. Values are represented as mean ± standard deviation

	RPOA	Dysplasia	OA	AVN
Superior joint space narrowing (mm/year)	2.44 ± 1.48	0.823 ± 1.08 (*P* = 0.019*)	0.765 ± 0.862 (*P* = 0.001*)	−0.584 ± 2.77 (*P* = 0.000*)
Superior joint space narrowing (percentage/year)	49.2 ± 29.7	11.9 ± 8.45 (*P* = 0.004*)	18.8 ± 21.2 (*P* = 0.003*)	−16.4 ± 76.2 (*P* = 0.003*)
Leg length shortening (mm/year)	15.7 ± 12.4	14.8 ± 27.0 (*P* = 0.902)	2.40 ± 2.27 (*P* = 0.001*)	6.02 ± 10.4 (*P* = 0.007*)
Superior acetabular bone loss (mm/year)	7.24 ± 3.69	3.06 ± 5.53 (*P* = 0.018*)	0.957 ± 1.32 (*P* = 0.000*)	2.21 ± 3.67 (*P* = 0.000*)
Acetabular volumetric widening (mm/year)	3.83 ± 5.33	2.08 ± 3.20 (*P* = 0.281)	0.459 ± 0.641 (*P* = 0.032*)	1.32 ± 2.92 (*P* = 0.044*)
Vertical head diameter change (mm/year)	15.2 ± 22.9	13.9 ± 27.4 (*P* = 0.884)	1.24 ± 1.49 (*P* = 0.031*)	6.40 ± 9.82 (*P* = 0.074)
Horizontal head diameter change (mm/year)	13.7 ± 21.8	6.29 ± 16.6 (*P* = 0.282)	1.05 ± 1.39 (*P* = 0.039*)	2.65 ± 6.33 (*P* = 0.012*)

*RPOA* Rapidly progressive osteoarthritis, *OA* osteoarthritis, *AVN* avascular necrosis

*: statistically significant

**Table 4:** The results validated using our proposed RPOA diagnostic criteria

	RPOA	Dysplasia	OA	AVN
Age > 65	Control	0.009^a^	0.694	0.000^a^
Pelvic tilt < 0.5		0.001^a^	0.032^a^	0.000^a^
Tonnis angle ≥5°		0.485	0.049^a^	0.001^a^
Superior joint space narrowing > 2 mm per year		0.070	0.021^a^	0.001^a^
Leg length shortening > 10 mm per year		0.285	0.001^a^	0.008^a^
Superior acetabular bone loss > 5 mm per year		0.004^a^	0.000^a^	0.000^a^
Acetabular volumetric widening > 5 mm per year		0.252	0.008^a^	0.020^a^
Head vertical diameter change > 5 mm per year		0.156	0.000^a^	0.031^a^
Head horizontal diameter change > 5 mm per year		0.282	0.004^a^	0.095

*RPOA* Rapidly progressive osteoarthritis, *OA* osteoarthritis, *AVN* avascular necrosis

^a^statistically significant

One single point was awarded to each parameter not described in literature: (1) changes in leg length discrepancy; (2) acetabular volumetric widening; (3) femoral head destruction; (4) Tonnis angle; and (5) lateral subluxation progression.

## Results

**Fig. 3:**
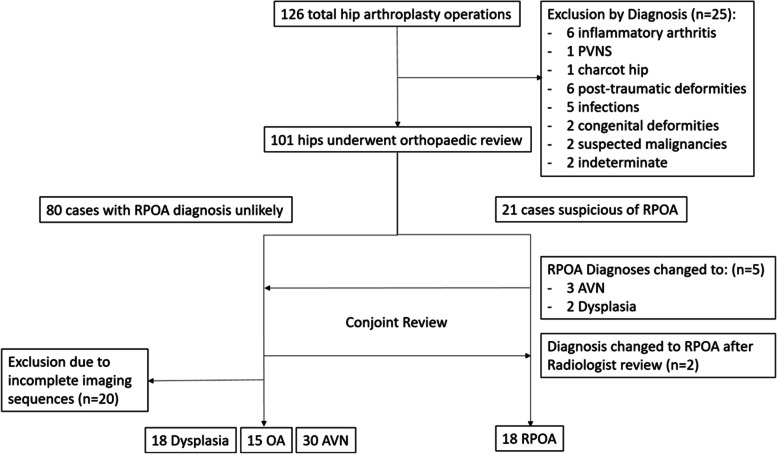
The flow diagram showing 126 pre operative images of hip arthroplasty operations reviewed. RPOA: Rapidly progressive osteoarthritis. OA: primary osteoarthritis. AVN: avascular necrosis. PVNS: pigmented villonodular synovitis

We recruited a total of 126 hips. Among them, 45 hips were excluded against the exclusion criteria. Following our review process (Fig. 3), we recruited 18 RPOA (incidence of 14%), 18 dysplasia, 15 OA and 30 AVN hips. Of the RPOA hips, 9 (50%) hips progressed from normal to complete obliteration of joint space, followed by femoral head and acetabular destruction. Two hips progressed from normal to type 1, and 4 (22%) hips from normal to type 2. Three (17%) hips were already at the stage of bony destruction upon the initial consultation. The average time from symptom onset to complete joint space obliteration was 19 months (range: 2 to 39 months). Based on Karayiannis’s classification [8], there were 8 rapid, 4 moderate, and 3 delayed chondrolysis hip RPOA. The history of symptom onset was undefined in the remaining 3 hips.

The RPOA group presented with older age, an initial lower leg length discrepancy, and higher final leg length discrepancy. A significantly greater Tonnis angle and posterior pelvic tilt was evident (Table 2). The rate of superior joint space narrowing, and subsequently the acetabular and femoral head bone loss in the RPOA group, differed significantly from those of OA and AVN groups. On the other hand, dysplastic hips showed comparable rate change in limb shortening and acetabular widening, but with the superior acetabular destruction being slower (Table 3).

As a higher Tonnis angle had been postulated in RPOA [10], its radiological progression was further evaluated. In our series, Tonnis angle increased from 8 to 14 degrees (*P* = 0.031), with yearly increase being 6.2 degrees per year. No significant changes were found in other groups (Dysplasia: 17 *vs*. 18, *P* = 0.494; OA: 6 *vs*. 7, *P* = 0.444; AVN: 4 *vs*. 4, *P* = 0.976). The rate change of lateral subluxation also progressed significantly in only RPOA (13 *vs*. 19, annual progression being 11 mm, *P* = 0.001) and dysplasia groups (20 *vs*. 23, annual progression being 7.6 mm, *P* = 0.034), but no progression took place in AVN and OA.

The RPOA, OA, and AVN scores assessed by different raters ranged 6.8–8.5, 1.9–2.0, and 1.5–1.6 respectively. Using a cutoff score of 5 points, 15 out of 18 RPOA met the RPOA diagnostic criteria, while 44 out of 45 non-RPOA hips did not fulfill the criteria. The percentage agreement of validating the diagnosis amongst all five raters was 95.6 and 99.6% in RPOA and non-RPOA groups, respectively, showing high inter-rater reliability. For individual scoring criteria, pelvic tilt diameter and superior acetabular bone loss had the highest and lowest percentage agreement respectively (Table 5). Using our patient series for validation, our cohort yielded a sensitivity of 83%, specificity of 98%, positive predictive value of 94%, and negative predictive value of 94% with the RPOA diagnostic criteria.

**Table 5:** Assessment of interrater reliability based on the RPOA diagnostic criteria

	Interrater percentage agreement
Pelvic tilt parameter	99%
Initial Tonnis angle	97%
Superior joint space narrowing	94%
Leg length shortening	96%
Superior acetabular bone loss	93%
Acetabular volumetric widening	94%
Femoral head destruction	96%
Tonnis angle progression	94%
Lateral subluxation progression	94%

The exact points of each patient scored were also compared amongst the raters. The intra-class correlation coefficient (ICC) for RPOA, OA, and AVN were 0.714, 0.883, and 0.873, respectively. The score calculation was repeated at a one-week interval, with the average intra-rater coefficient being 0.965.

## Discussion

RPOA is classified into type 1 and type 2 in terms of the absence or presence of bony destruction [6, 7]. Karayiannis et al [8] described another classification system on the basis of the time interval and rate of bone loss (type 1, rapid; type 2, moderate; and type 3, delayed). The patterns of disease progression varied in our study. It is difficult to recognize transition from type 1 to type 2 RPOA due to the lack of clinical signs and proper serial imaging.

Our RPOA diagnostic criteria exclude hip dysplasia from the major components of criteria because dysplasia is difficult to be differentiated from RPOA at the early stage of chondrolysis and the late stage of bony destruction [7]. Thus, our diagnostic criteria emphasize the importance to rule out dysplasia from the standard definitions [9]. Our criteria include both initial imaging and progression assessments. When the hip is still grossly intact, a higher Tonnis angle and lower pelvic tilt parameter predict the development RPOA in the future and differentiate this condition from dysplasia [7] and osteonecrosis [10]. It is in line with our results and the existing literature.

Subsequent imaging provides evidence to assess the rate of cartilage and bony destruction. In our study, new radiological findings were found and were in concordance with the proposed pathophysiology of bony destruction and supero-lateral wear. Changes in leg length discrepancy, acetabular volumetric widening, femoral head destruction, Tonnis angle and lateral subluxation progression, all demonstrated statistically significant differences between RPOA group and non-RPOA groups in our study. However, these parameters have not been described in the existing literature. Both superior bone loss and widening contribute to acetabular bone deficit, and femoral head destruction reduces autograft available for acetabular reconstruction.

Dysplastic hips showed a similar rate of length shortening, volumetric widening and head destruction. The two conditions can be distinguished clinically in light of younger-age onset, initial radiographic films showing a higher Tonnis angle of more than 10 degrees, and lower Wiberg angle of less than 25 degrees in the dysplastic group. In the AVN group, although early head destruction is common, the condition can present with head flattening and paradoxical joint widening, unlike RPOA, which manifests as early joint space obliteration.

Until now, the etiology of RPOA has not been well understood. The proposed mechanisms include idiopathic chondrolysis, subchondral-insufficiency fracture, labrum inversion or immunological reaction with activation of osteoclasts [11]. Recently, some surgeons proposed that the abnormal anatomical and morphological factors might underlie rapid progressive antero-superior and supero-lateral wear [12], including increased Tonnis angle, Wiberg angle, acetabular extrusion index [10], and abnormal pelvic tilt [7]. As opposed to decreased dysplasia parameters in elderly osteoarthritis [13], an abnormally high Tonnis angle may constitute a different mechanism, leading to rapidly progressive osteoarthritis. On the sagittal plane, pelvic tilt has also been proposed as a potential causative mechanistic factor [14]. With an abnormal posterior pelvic tilt secondary to degenerative lumbar kyphosis, bone destruction commenced in the anterior portion of the femoral head in all type 2 patients [7]. This consequence leads to wear in the antero-superior portion of the acetabulum, in coincidence with the description by Thompson *et al* [12] and Karayiannis *et al* [8]. This finding has intraoperative significance, since a large antero-superior gap will be anticipated during acetabular reaming. The acquired deformity of Tonnis angle and lateral subluxation progression may further decrease hip conformity and containment, aggravating lateral edge wear and resulting in acetabular and head destruction. This may explain why the subsequent bony loss is usually rapidly progressive. Our postulation also explains the observation that bony destruction in RPOA is usually supero-lateral as well as supero-anterior [12].

Our study has limitations. First, the patients were recruited only in the Southeast Asian population, and the results may not be generalized to other populations. Second, the retrospective nature and small sample size might result in confounding bias. Third, the intervals of subsequent imaging and the waiting time to operation varied widely due to rapid clinical progression and the patient’s choice for operation. We couldn't obtain the images of different patients at exactly the same time intervals. Fourth, we only included the patients on the fast-track hip replacement list with complete imaging sequences, possibly resulting in selection bias. Fifth, most patients with RPOA underwent an operation early, but some OA patients may be put on further observation. This led to shorter follow-up duration in the former group. Sixth, the bony landmarks may be destructed due to RPOA, increasing measurement variations compared to non-RPOA conditions. Seventh, it might be difficult to measure the Wiberg angle due to gross head destruction and the loss of head center. Finally, volumetric acetabular width was only estimated based on 2-dimensional length instead of 3-dimensional volumetric measurement, which may not reflect the entire situation of acetabular erosion.

## Conclusions

Posterior pelvic tilt and increased Tonnis angle may be involved in the pathogenesis of RPOA, leading to progressive acquired acetabular obliquity and lateral subluxation. We propose the modern comprehensive diagnostic criteria be based on the existing literature and our current findings. Further external validation is recommended.

## Data Availability

not available

## Abbreviations

RPOA: Rapidly progressive osteoarthritis
AVN: Avascular necrosis
OA: Primary osteoarthritis
